# Infectious Diseases Seeker (IDS): An Innovative Tool for Prompt Identification of Infectious Diseases during Outbreaks

**DOI:** 10.3390/ijerph18063216

**Published:** 2021-03-20

**Authors:** Federico Baldassi, Mariachiara Carestia, Stefania Moramarco, Andrea Malizia, Pasquale Gaudio

**Affiliations:** 1 Department of Industrial Engineering, University of Rome “Tor Vergata”, 00133 Rome, Italy; gaudio@ing.uniroma2.it; 2 Department of Biomedicine and Prevention, University of Rome “Tor Vergata”, 00133 Rome, Italy; mariachiara.carestia@uniroma2.it (M.C.); stefania.moramarco@gmail.com (S.M.); malizia@ing.uniroma2.it (A.M.)

**Keywords:** Infectious Diseases Seeker (IDS), epidemiology, emerging and re-emerging infectious diseases, pathogens, outbreaks, public health, yellow fever, COVID-19

## Abstract

Background: Several technologies for rapid molecular identification of pathogens are currently available; jointly with monitoring tools (i.e., web-based surveillance tools, infectious diseases modelers, and epidemic intelligence methods), they represent important components for timely outbreak detection and identification of the involved pathogen. The application of these approaches is usually feasible and effective when performed by healthcare professionals with specific expertise and skills and when data and resources are easily accessible. Contrariwise, in the field situation where healthcare workers or first responders from heterogeneous competences can be asked to investigate an outbreak of unknown origin, a simple and suitable tool for rapid agent identification and appropriate outbreak management is highly needed. Most especially when time is limited, available data are incomplete, and accessible infrastructure and resources are inadequate. The use of a prompt, user-friendly, and accessible tool able to rapidly recognize an infectious disease outbreak and with high sensitivity and precision may be a game-changer to support emergency response and public health investigations. Methods: This paper presents the work performed to implement and test an innovative tool for prompt identification of infectious diseases during outbreaks, called Infectious Diseases Seeker (IDS). IDS is a standalone software that runs on the most common operative systems. It has been built by integrating a database containing an interim set of 60 different disease causative agents and COVID-19 data and is able to work in an off-line mode without requiring a network connection. Results: IDS has been applied in a real and complex scenario in terms of concomitant infectious diseases (yellow fever, COVID-19, and Lassa fever), as can be in the second part of 2020 in Nigeria. The outcomes have allowed inferring that yellow fever (YF), and not Lassa fever, was affecting the area under investigation. Conclusions: Our result suggests that a tool like IDS could be valuable for the quick and easy identification and discrimination of infectious disease outbreaks even when concurrent outbreaks occur, like for the case study of YF and COVID-19 pandemic in Nigeria.

## 1. Introduction

Infectious diseases, due to emerging and reemerging pathogens, are a cause for concern. Besides COVID-19, a plethora of other viral pathogens such as Ebola, type A influenza (swine flu and bird flu), Lassa, yellow fever (YF), dengue, and West Nile virus, can be found in nature and specifically in some regions of the world, namely Western and Central Africa. These pathogens are responsible for high morbidity and mortality in humans, and most of them can spread rapidly and cause severe epidemics and pandemics among the human population within a short time period, covering a broad geographical area [1]. 

An outbreak is defined as the occurrence of disease cases exceeding normal expectancy in a specific location over a specific period of time. Specific tools spanning from molecular assays for the identification of pathogens to monitoring tools (i.e., web-based surveillance tools, infectious diseases modelers, and epidemic intelligence methods) all represent important components for timely outbreak detection and for the identification of the involved pathogen [2]. Suspicion often arises when healthcare workers report an unusual cluster or a single, unexpected presentation. However, this surveillance may cause a delay in the detection of an outbreak [3], especially in those areas where public health and epidemic surveillance systems are scarcely present or have limited resources. 

Moreover, these laboratory and epidemiological investigations, despite being effective and reliable, need to be performed by professional users with a specific scientific background; besides, a sufficient amount and quality of input data and time resources are usually required and, in many circumstances, access to web resources is required.

Contrariwise, in field situations or during operations in remote and underserved areas where available data are incomplete and infrastructure and resources are inadequate, first responders and healthcare workers might benefit from an accessible and easy-to-use tool for rapid discrimination of infectious disease outbreaks, most especially if considering that the first responders with heterogeneous competences and various levels of knowledge may need to face an outbreak of unknown origin with limited time for action. This could immediately have a positive impact on the time needed to address an emergency response and support an effective and more structured public health response. The use of a prompt, user-friendly, and accessible tool able to rapidly recognize the infectious disease that may possibly result in an outbreak, and with high sensitivity and precision, can be paramount to support the subsequent outbreak investigation and related public health measures. To the best of our knowledge, no decision support tool exists for the rapid and inexpensive assessment of outbreaks, especially when available information and resources are limited [4].

This paper presents the work performed to set up and implement a standalone software, called Infectious Diseases Seeker (IDS), developed in the MATLAB^®^ environment. One of the strengths of IDS is that it runs on the most common operating systems (Windows, Linux^®^, and Mac) and does not require a licensed copy of MATLAB^®^. Moreover, it was built by integrating a database containing distinctive data of an interim set of 61 different disease causative agents and, consequently, is able to work in an off-line mode without requiring a network connection. In this work, we also tested IDS in a real and complex scenario, i.e., Nigeria, where the fast identification of an outbreak is pivotal for the early management of such possible public health threats. 

## 2. Materials and Methods

### 2.1. Setting

Nigeria is a high-risk country experiencing multiple and recurrent outbreaks [5]. The reemergence of YF in September 2017 in Nigeria has been marked by outbreaks over a wide geographical area. From August to November 2019, the YF outbreaks reported in Bauchi, Benue, and Katsina states with spread to multiple other states showed an expansion of YF transmission and an elevated risk for the outbreak to rapidly spread and amplify, impacting areas without prior reported cases since 2017 as well as areas with large under-immunized populations. In the second part of 2020, suspected YF cases were reported from all 36 states, and confirmed YF cases were reported across nine states (Delta, Enugu, Bauchi, Benue, Kogi, Oyo, Edo, Kwara, and Katsina). These new outbreaks in Bauchi, Delta, and Enugu are affecting areas without prior reported cases this year and suggest underlying sustained high viral transmission in the epizootic cycle with a spillover to human populations. Due to the risk of diffusion to neighboring countries with under-immunized populations, including to large urban centers, the high CFR, the potential for ongoing local transmission and amplification due to suboptimal vaccination coverage, and the occurrence of cases in peri-urban areas (e.g., in Delta State) and densely populated local government areas, the risk is extremely high [6]. In 2020, Nigeria faced concurrent outbreaks of multiple pathogens, such as YF and Lassa fever viruses, in addition to COVID-19, resulting in a severe overload of the country’s health system [5,6,7,8,9,10].

### 2.2. Database

The key to this software being useful and workable in practice is the quality and reliability of the database on which the predictions are based. The database was populated with the relevant data of 61 pathogens and the relative infectious diseases they cause and, the period of data collection spans from January 2019 to October 2020.

The database of the first prototype, presented for the first time [11,12,13], has been developed based on the current landscape of publicly available data on infectious disease outbreaks. The database was built using data reported through several sources, namely: World Health Organization (WHO)/United Nations (UN), U.S. Centers for Disease Control and Prevention (CDC), European Centre for Disease Prevention and Control (ECDC), Global Burden of Disease (GBD), Program for Monitoring Emerging Disease (ProMED), scientific literature [14,15,16,17,18].

The list was not fully exhaustive, and it did not include pathogens that cause rare infectious diseases. The list was built by selecting different categories of pathogens (viruses, bacteria, and parasites) to cover the most significant infectious diseases, potentially resulting in high impact consequences for public health as much as possible. 

The epidemiological parameter selection for each pathogen was derived from an exhaustive review of some of the most relevant scientific papers existing in literature and other related sources (e.g., WHO, CDC, or ECDC official websites, reports, and publications) and in accordance with the outcome of a survey performed at international level. The selection allowed describing, as well as possible, the main characteristics of each agent and its related disease; in fact, the goal was to define, unequivocally with refined parameters, each pathogen that had been taken into account to help the software in its searching and prediction process. 

A database string was composed of the parameters as shown in Table 1 [13].

### 2.3. Infectious Diseases Seeker (IDS)

IDS was thought of and developed to make it as scalable as possible. At this level of development, for instance, the user can use just one of the five functions of the software available or combine them, depending on the user’s needs, in diverse combinations with the capabilities of two or more IDS functions (see Section 3).

**Figure 1:**
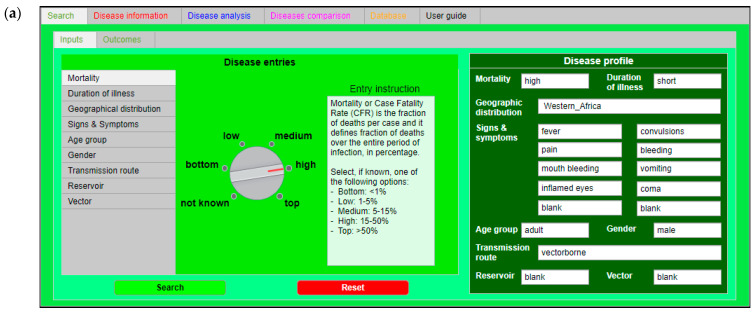

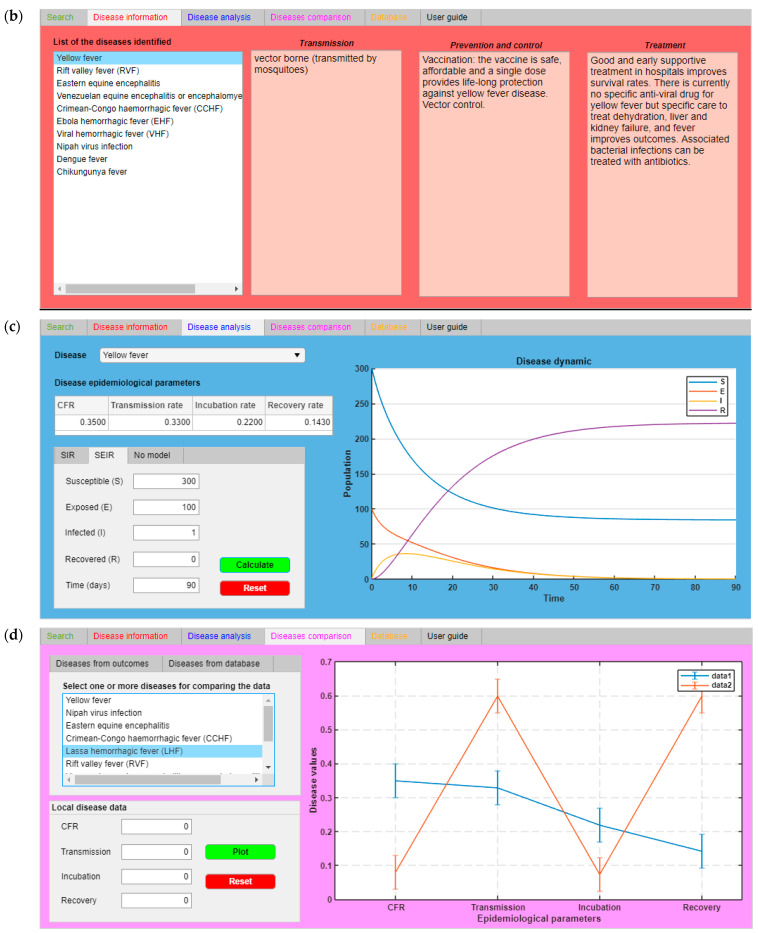

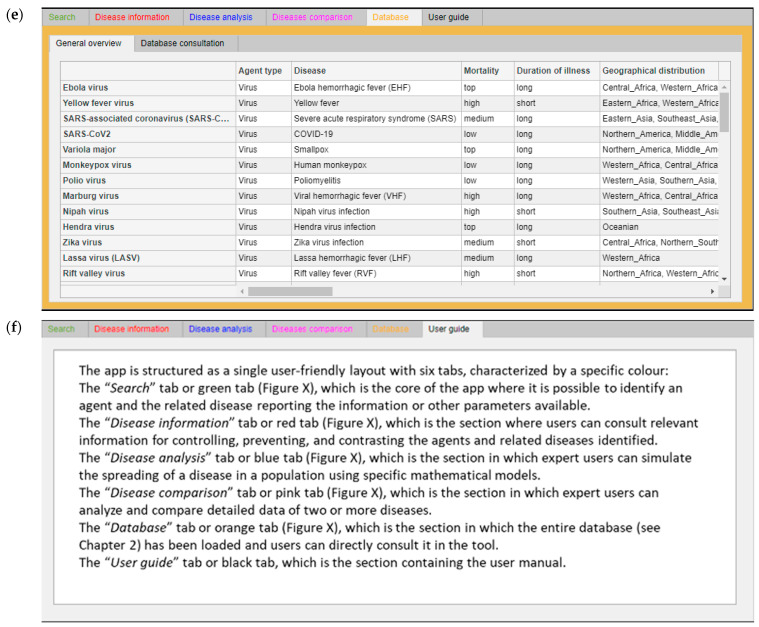
IDS screenshots of the six tabs. (**a**) “Search” tab or green tab; (**b**) “Disease information” tab or red tab; (**c**) “Disease analysis” tab or blue tab; (**d**) “Disease comparison” tab or magenta tab; (**e**) “Database” tab or orange tab; and (**f**) “User guide” tab or black tab.

IDS was designed as an intuitive tool with a user-friendly layout with six tabs, each characterized by a specific color [13]. Figure 1 refers to the YF epidemic analysis and provides the input and output data for the case study while showing the user interface.

Each IDS tab is briefly described below:

1. The “Search” tab (Figure 1a) is the core of IDS: Here, the identification of an agent and the related disease is performed. These parameters, inserted in the “Inputs” sub-tab, have been selected as they are considered the most significant factors, that even a non-healthcare worker can identify and recognize. It is possible to load these data in the “Disease parameters” section as free text values, using a drop-down menu or switchers, following the instructions reported in the “Parameter instruction” frame of each parameter sheet. The input loaded in this tab is summarized in the “Disease profile” section. IDS works with a regressive analysis (logistic regression analysis) that is able to determine whether data loaded by the user in the “Disease profile” section are in the database datasets and then recognize the associated correspondences and the related accuracy ratio [12,13].
2. The “Disease information” tab (Figure 1b) is an IDS function: Here, users can consult relevant and appropriate information for controlling, preventing, and contrasting the agents and related diseases identified. This tab is divided into three sections:
  - “Transmission” section: General information on how agent/disease can be transmitted.
  - “Prevention and control” section: General information that are suggestions and recommendations against an agent and its related disease.
  - “Treatment” section: Information on what kind of medical treatment is available for an agent and its related disease.
  The red tab is significant for non-professional users, in particular, in the case of emergencies. Once the possible causative agent is identified, the user can read the information of this section to take early measures and reduce the spreading of the infectious disease.
3. The “Disease analysis” tab (Figure 1c) is the section in which expert users can simulate the spreading of a disease in a population using one specific compartmental model, either the Susceptible-Infected-Recovered (SIR) or Susceptible-Exposed-Infected-Recovered (SEIR) models [19].
4. The “Disease comparison” tab (Figure 1d) is the section in which expert users can analyze and compare other specific epidemiological parameters (CFR, transmission rate, incubation rate, and recovery rate) of two or more diseases. This tab is particularly useful when an end-user has to compare the local data, coming from a real case, and the data loaded in the IDS database.
5. The “Database” tab (Figure 1e) is the section in which the entire database has been loaded and users can directly consult it in the tool. The database has two sections: a general overview of the database and a section where it is possible to consult, search, and classify specific records.
6. The “User guide” tab (Figure 1f) is the section containing the user manual.

### 2.4. Scenario

The actual scenario that was tested and investigated in this paper was the YF outbreak in Nigeria in 2020. There were two main reasons for this: (i) the poor available information on this disease provided by the local authorities, and (ii) the concurrent public health emergencies, such as measles, monkeypox, Lassa fever, and COVID-19 in the same area or country.

Table 2 summarizes the data obtained from the WHO report of 24 November 2020, “Disease outbreak news” [6]. This document reported a cluster of deaths from an undiagnosed disease were notified on 1 November 2020 through Event-Based Surveillance in two states, Delta and Enugu, located in southern Nigeria. Moreover, the Delta State health surveillance system had been informed of the outbreak on 30 October 2020 following a cluster of deaths that occurred in the presence of similar symptoms. The suggestive differential diagnosis was Lassa fever, YF, cerebrospinal meningitis, and COVID-19. 

## 3. Results

**Figure 2:**
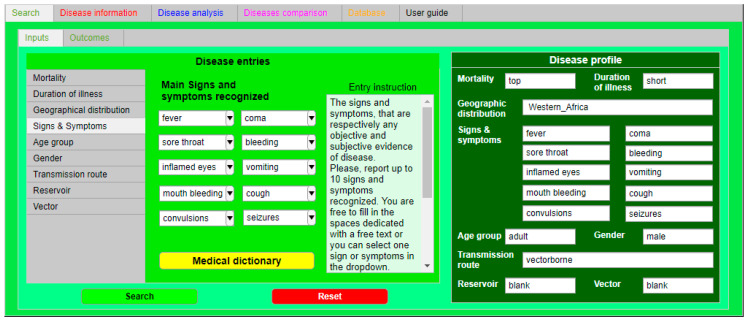
“Search” tab screenshot. “Search” tab layout after the loading of data available for YF. On the right in dark green, the “Disease profile” section, where it is possible to check and inspect all the YF data filled in.

To test the capabilities of IDS in predicting and dealing with a real and complex scenario, as previously described in Section 2.2, the available data of the YF outbreak, summarized in Table 2, were loaded into IDS (Figure 2).

**Figure 3:**
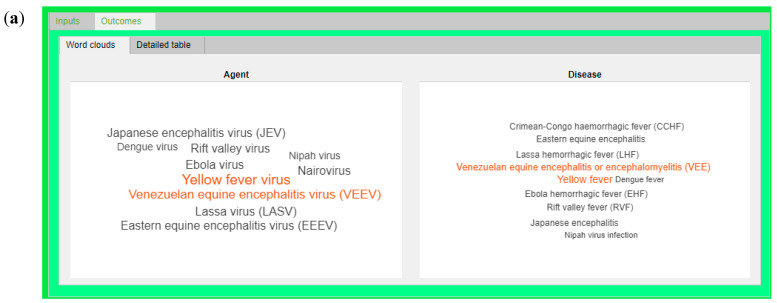

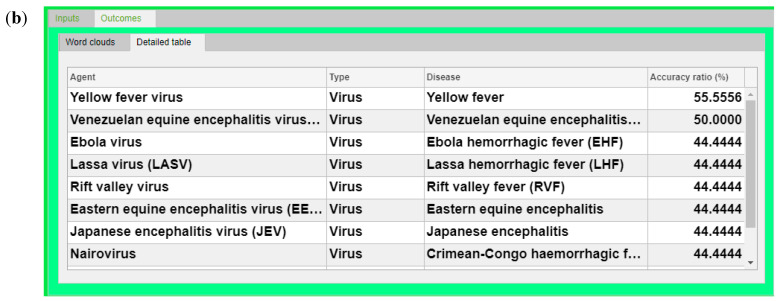
IDS outcomes screenshots. (**a**) Word cloud plot and (**b**) detailed table.

Once IDS elaborated the input data, the outcomes concerning the software identification are represented, as described below:

1. As a word cloud plot (Figure 3a). This plot can be used for an easy first look, in fact, each word cloud plot is a visual representation of the IDS outcomes and the size and color of each disease identified, indicating its relative accuracy ratio. In this case, the YF virus, the Venezuelan equine encephalitis virus, and their related disease proved to be the most likely cause for the outbreak.
2. As a detailed table (Figure 3b). This outcome representation shows the accuracy ratio of the identification expressed in percentage and calculated as a summary quantitative measure of matched values between data availability and the database [12,13]. As shown in the table, the YF virus and its related disease were reported in the first position with an accuracy ratio of 55.5%.

**Figure 4:**
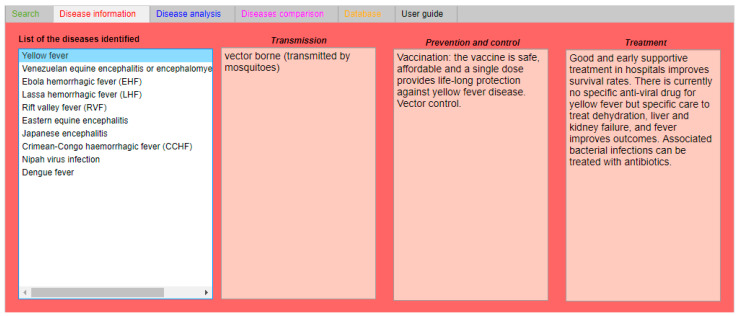
“Disease information” tab showing the YF information on transmission, prevention and control, and treatment.

Once the 10 closest matching results have been identified, the “Disease information” tab provides the user with essential information for controlling and preventing the spread of the predicted outbreak’s causative agent, as shown in Figure 4. For instance, taking into consideration the “Prevention and control” section, IDS suggests a vaccination and vector control process for controlling the outbreak, in accordance with the WHO guidelines for YF [14].

**Figure 5:**
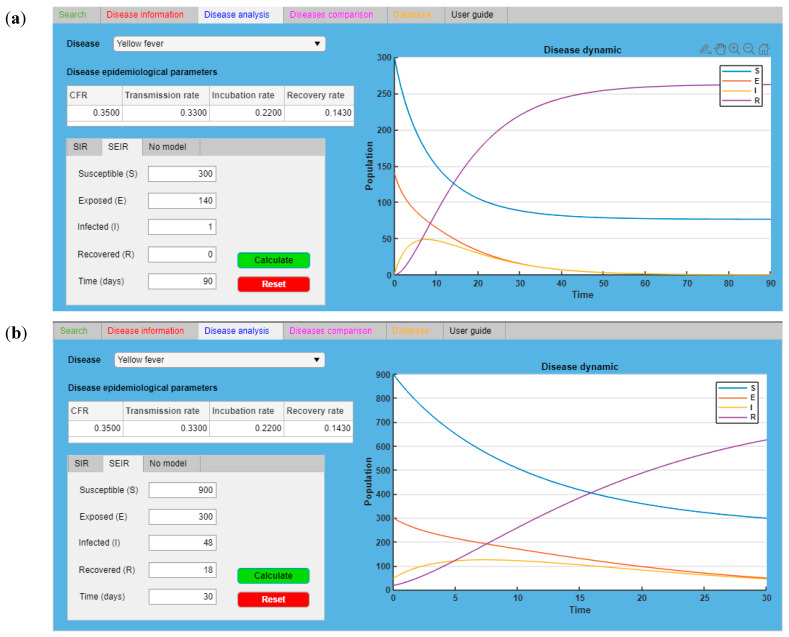
“Disease analysis” table showing (**a**) The YF dynamic in Delta state (Nigeria) from the index case (28 July 2020) to 4 November 2020: real data confirmation. (**b**) The forecast of YF dynamic in Delta State (Nigeria) from 4 November 2020 to the next 30 days.

By applying the “Disease analysis” tab of IDS, it was possible to evaluate the diffusion dynamics of YF. In this tab, the user, operating on the drop-down button and selecting “Yellow fever”, automatically obtained the related epidemiological parameters and the mathematical model connected to this disease (SEIR). Once the fields “Susceptible (S)”, “Exposed (E)”, “Infected (I)”, Recovered (R)”, and “Time (days)” had been filled with the input data (here, taking into account the local data coming from the Delta state [6] in Nigeria), it was possible to follow the YF trend from the index case to the next 90 days (from 24 July 2020 to 4 November 2020). Figure 5a shows and confirms the actual dynamic of YF in Delta state. In this simulation, the people in S and E classes were estimated. 

Once the data were confirmed by using the same function and model, it was possible to predict, with the proper assumptions, the future trend of YF from 4 November 2020 to the next 30 days (Figure 5b). The user can select the period of time desired, but it is important to consider that the level of confidence can decrease due to the increase in time. Moreover, the individuals in S and E classes were estimated.

**Figure 6:**
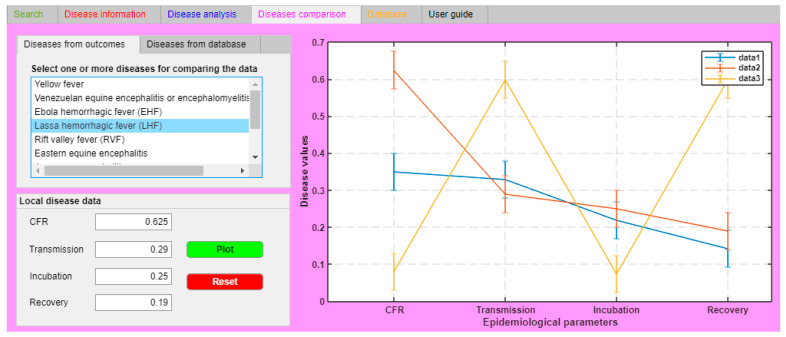
“Disease comparison” tab screenshot. The correspondence between YF database data (blue line—data1), YF local data (red line—data2), and Lassa fever database data (yellow line—data 3). The CFR in the “Local disease data” section is in decimals, and Transmission, Incubation, and Recovery are in day^−1^.

The last set of processed data is available in the “Disease Comparison” tab (in magenta). This function allows comparison of specific epidemiological data between those available in the database and field data. Field data were calculated based on data available from WHO reports [7]. Specifically, the CFR was 62.5% (0.625 in decimals), the transmission was 0.29 day^−1^ (3.5 days), the incubation was 0.25 day^−1^ (4 days), and the recovery was 0.19 day^−1^ (about 5.5 days). As shown in Figure 6, the local YF data matched database data related to YF with an estimated accuracy of 75% (3 out of 4 parameters matched), while there was no correlation between the local YF and database data regarding Lassa fever (0 out of 4 parameters matched). This operation was performed because, as the WHO reports, the differential diagnosis did not allow a distinction between Lassa fever and YF. This other type of IDS analysis allowed us to identify visually and quite clearly, that the area under investigation was probably affected by YF and not Lassa fever. In addition, this analysis confirmed what had been obtained through the application of the “Search” function, i.e., the identification of YF in the involved area.

## 4. Discussion

As WHO reports, Nigeria is facing concurrent outbreaks of multiple pathogens, including COVID-19 [5,6,7]. National and state authorities are currently focused on the COVID-19 pandemic, limiting the human resources required to conduct investigations and response activities for other outbreaks, such as YF. COVID-19 response efforts demand an extraordinary amount of time and resources from the country’s health system while lockdown, travel restrictions, and other mitigation to slow the spread have severely disrupted access to core essential health services [8,9,10]. 

Failure in the surveillance of infectious diseases in this rapidly changing context exposes health systems to critical gaps in services and lack of information when they are most needed and can have a long-lasting impact on the health and wellbeing of populations [20].

Suspicion often arises when healthcare workers report an unusual cluster or a single, unexpected event. This passive surveillance leads to a delay in the detection of an outbreak. To address these issues, specific tools, such as online databases and surveillance-reporting networks, have been developed to identify and monitor the emergence and spread of infectious agents. These include tools to aid in the clinical diagnosis of a single case of infectious diseases, tools that process unverified epidemic intelligence using specific keywords, e.g., HealthMap.org and Google Flu Trends, those that compile verified outbreak data, e.g., Global Early Warning System for major animal diseases including zoonoses (GLEWS), Global Animal INformation System (GAINS), Global Infectious Disease and Epidemiology Network (GIDEON), and those that disseminate expert-moderated outbreak reports and anecdotal information, e.g., ProMED-mail [14,15,16,17,18].

Moreover, to the best of our knowledge, no decision support tool exists for the rapid and inexpensive assessment of outbreaks, particularly when faced with minimal information and limited resources [4].

IDS has been tested on the YF outbreak in Nigeria in 2020 to evaluate the ability of IDS to discriminate against a given outbreak with respect to the existing database. Considering a context, like the one analyzed in this work, where many emerging and reemerging biological threats were present and the healthcare surveillance system was jeopardized by multiple threats and scarce resources, having additional easy-to-use tools to detect infectious disease outbreaks can be extremely useful. Here, we propose that the application of IDS by non-expert users, such as first responders or non-healthcare workers, can be effective in identifying the YF outbreak in Nigeria and provide some relevant information for controlling and preventing further spread of the disease. In addition, application of IDS by expert users, such as healthcare workers, has been useful for understanding the possible dynamic of YF in the population and predicting the probable future trend in the considered region. Moreover, expert users are able to obtain additional data that confirm, in this case, the previous YF identification by the comparison of more specific epidemiological data coming from local information with the data loaded in the database.

Limitations related to the scarcity of input data must be fully investigated, and validation for other sources of outbreaks of infectious diseases must be performed, especially to assess the robustness of IDS when a less or more complete dataset is available. An intrinsic methodological limitation of IDS is related to the database and the fact that only diseases are included. To reduce this limitation, we intend to expand the database with additional infectious disease parameters and to test the related effect on the specificity and sensitivity of IDS.

Concerning the use of IDS, it is intended for the use of users with little to no epidemiological background; therefore, the next step will be to perform user experience tests and investigate possible variables of the robustness of the tool when used by different types of users.

Furthermore, it is our intention to improve specific tool functions such as the “Disease analysis” tab. To do this, an idea could be to add a wide selection of specific countermeasures (isolation, quarantine, social distancing, and vaccination) to the pre-loaded mathematical models (SIR and SEIR) to study and analyze the spread of infectious diseases with respect to these additional variables. 

In addition, a future objective of the developers of IDS, in accordance with the outcomes from the previous studies and this paper, will be to further analyze the needs of different types of users and customize IDS accordingly. 

Finally, the optimization and application of IDS to different risk areas, like that of food security and safety [21], health and humanitarian priority when talking about fragile countries, and remote and underserved populations, will be investigated in the future.

## 5. Conclusions

IDS can represent a valuable, user-friendly tool for a first fast and effective response to suspected infectious disease outbreaks, especially where the availability of epidemiological tools and specific knowledge is limited. This could positively impact all public health measures that aim for the prevention or distinguishing of infectious diseases, especially in remote or underserved communities or in case of emergencies. In this work, we showed the working principle and developed software, testing it in an actual setting showing that IDS was able to detect and discriminate the occurrence of a YF outbreak concurrently with the COVID-19 pandemic and other outbreaks, such as Lassa fever, occurring in Nigeria in 2020. 

This represents a firm step toward the further integration of the database, a validation with more different case studies, and the design of a more user-friendly interface.

## Figures and Tables

**Table 1:** Example of a database string. * A 95% Confidence Interval (95% CI) for all the detailed numeric parameters have been considered (CFR, Transmission rate, Incubation rate, Recovery rate, Infectious mortality rate). ** Susceptible-Infected-Recovery (SIR) mathematical model describes how individuals move through each compartment in the model.

Agent Parameters	Value
Agent name	Lassa virus (LASV)
Agent type	Virus
Disease	Lassa hemorrhagic fever (LHF)
Mortality	Medium
Duration of illness	Long
Geographical distribution	Western Africa
Signs & symptoms	Tiredness, headache, sore throat, muscle pain, chest pain, nausea, vomiting, diarrhea, cough, abdominal pain, facial swelling, fluid in the lung cavity, mouth bleeding, nose bleeding, vagina bleeding, gastrointestinal bleeding, low blood pressure
Age group	Baby, child, teenager, young, adult, senior
Gender	Male, female
Transmission route	Foodborne, contaminated surface
Reservoir/host/source	Rodent
Vector/other	None
Transmission	Via contact with food or household items contaminated with rodent urine or feces
Prevention and control	Promoting good “community hygiene” to discourage rodents from entering homes. Effective measures include storing grain and other food stuffs in rodent-proof containers, disposing of garbage far from the home, maintaining clean households, and keeping cats. Because *Mastomys* rodents are so abundant in endemic areas, it is not possible to eliminate them from the environment. Family members should always be careful to avoid contact with blood and body fluids while caring for sick persons.
Treatment	The antiviral drug ribavirin is an effective treatment for Lassa fever if given early on in the course of clinical illness. There is no evidence to support the role of ribavirin as post-exposure prophylactic treatment. There is currently no vaccine
CFR (decimals)	0.08 *
Transmission rate (day 1)	0.6 *
Incubation rate (day 1)	0.074 *
Recovery rate (day 1)	0.6 *
Infectious mortality rate (day 1)	0.2 *
Compartmental model	Susceptible-Infected-Recovery (SIR) **

**Table 2:** Data taken from the WHO report of 24 November 2020.

Data Available	Value
Index case	24 July 2020
Duration of illness	5 days
Location	Nigeria (Western Africa)
CFR	62.5%
Gender	Male
Age	4–65 years old
Signs and symptoms	Fever, vomiting (with or without blood), bleeding, mouth bleeding, convulsions, seizures, unconsciousness (coma), cough, sore throat, inflamed eyes
Transmission	Vector-borne (all people affected were farmers)

## Data Availability

Not applicable.
